# Supplementary material for: Exacerbated fires in Mediterranean Europe due to anthropogenic warming projected with non-stationary climate-fire models
Source: Nat Commun. 2018 Oct 2;9:3821. doi: 10.1038/s41467-018-06358-z (PMC6168540; doi:10.1038/s41467-018-06358-z)
Supplement: Supplementary file 1 — Supplementary Information [file 41467_2018_6358_MOESM1_ESM.pdf]

SUPPLEMENTARY INFORMATION
Exacerbated fires in Mediterranean Europe due to anthropogenic warming projected with non-stationary climate-fire models

Marco Turco[1,*], Juan José Rosa Cánovas[2], Joaquín Bedia[3,4], Sonia Jerez[2], Juan Pedro Montávez[2], Maria Carmen Llasat[1], and Antonello Provenzale[5]

[1]Department of Applied Physics, University of Barcelona, 08028 Barcelona, Spain
[2]Regional Atmospheric Modeling Group, University of Murcia, 30100 Murcia, Spain
[3]Predictia Intelligent Data Solutions, 39005 Santander, Spain
[4]Santander Meteorology Group. Department of Applied Mathematics and Computing Science, University of Cantabria, 39005, Santander, Spain
[5]Institute of Geosciences and Earth Resources (IGG), National Research Council (CNR), 56124 Pisa, Italy
[*]Corresponding Author turco.mrc@gmail.com

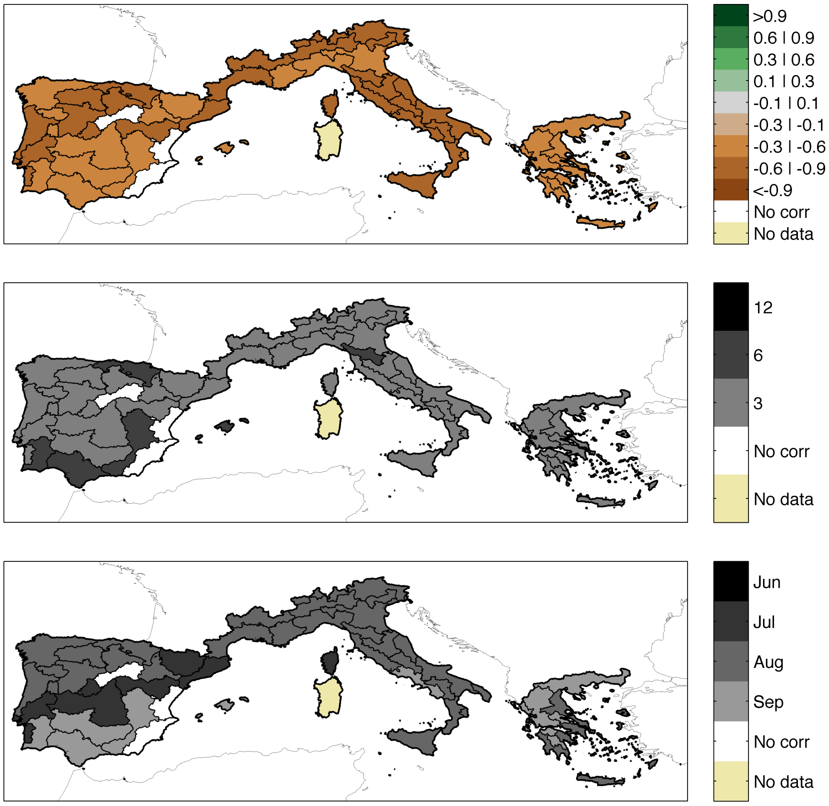

Supplementary Figure 1: Maximum significant correlation (in absolute value) between $\log(BA)$ and SPEI (first line); length of the period (3, 6 and 12 months; second line) and final month of accumulation of the SPEI for which the absolute value of the correlation is maximum (third line). Only correlations that are collectively significant from a False Discovery Rate test[1] are shown.

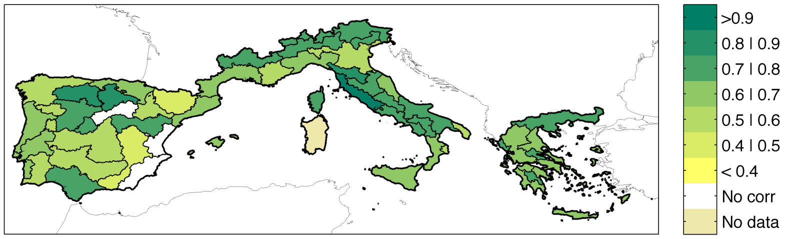

Supplementary Figure 2: Correlation between modelled and observed $\log(BA)$ for each eco-region.

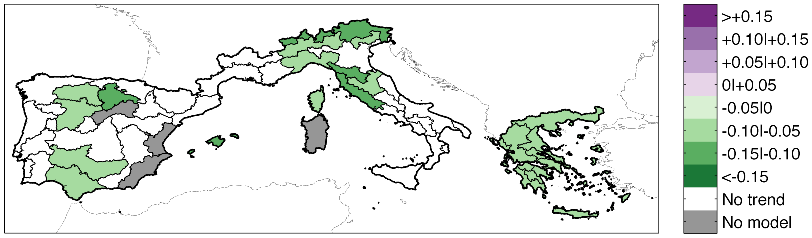

Supplementary Figure 3: Coefficient weights for the predictor time trend (i.e. the coefficient β_3 of Eq. 1 in the main paper).

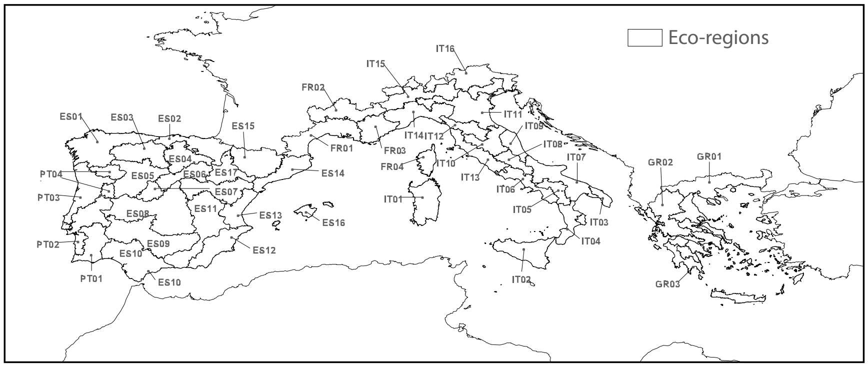

Supplementary Figure 4: Eco-regions and relative codes used in this study. See[2] and[3] for more details.

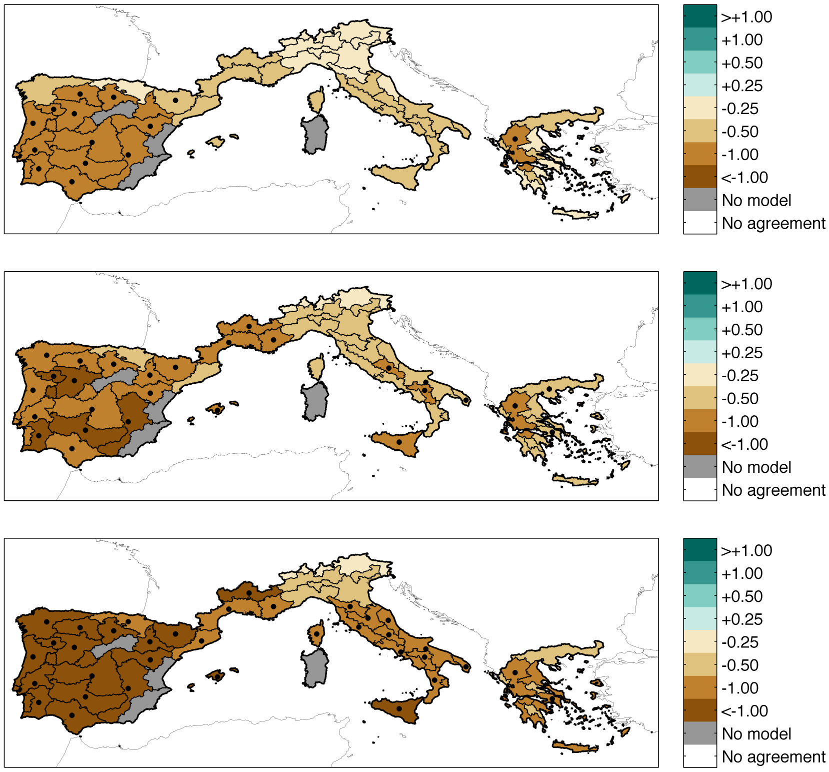

Supplementary Figure 5: Ensemble mean $SPEI_{sc}(m)$ (exact values of sc and m are available in Table 1) changes, with respect to 1971-2000, for the +1.5°C case (first line), the +2°C case (second line) and the +3°C case (third line). Dots indicate areas where at least 50% of the models show a statistically significant change and more than 66% agree on the direction of the change; coloured area (without dots) indicate that changes are small compared to natural variations; white regions (if any) indicate that no agreement between the RCMs is found (similarly to[4]).

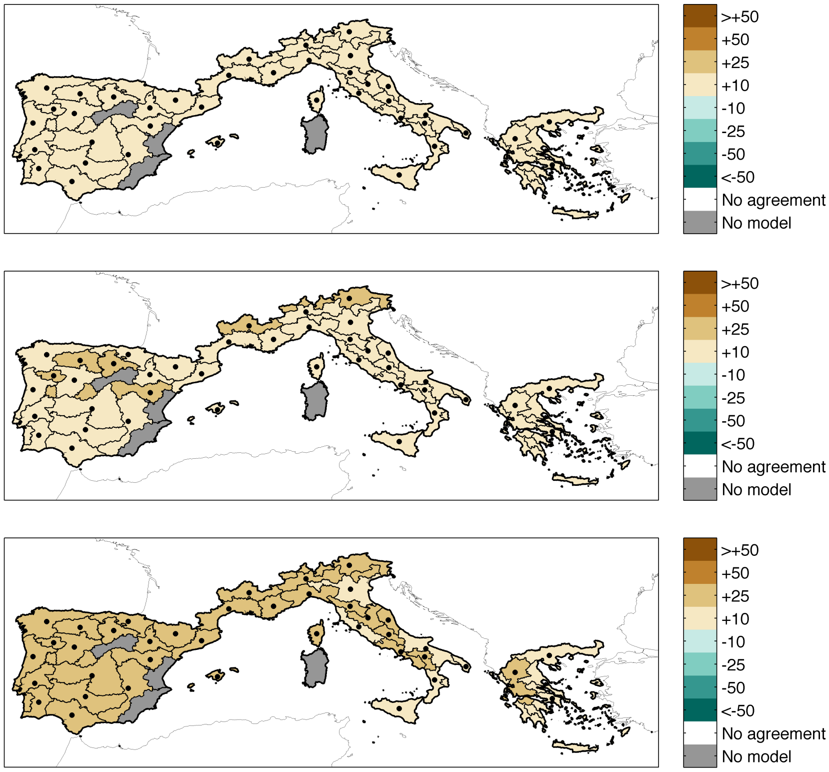

Supplementary Figure 6: Ensemble mean PET changes (in percentage), with respect to 1971-2000, for the +1.5°C case (first line), the +2°C case (second line) and the +3°C case (third line). Dots indicate areas where at least 50% of the models show a statistically significant change and more than 66% agree on the direction of the change; coloured area (without dots) indicate that changes are small compared to natural variations; white regions (if any) indicate that no agreement between the RCMs is found (similarly to[4]).

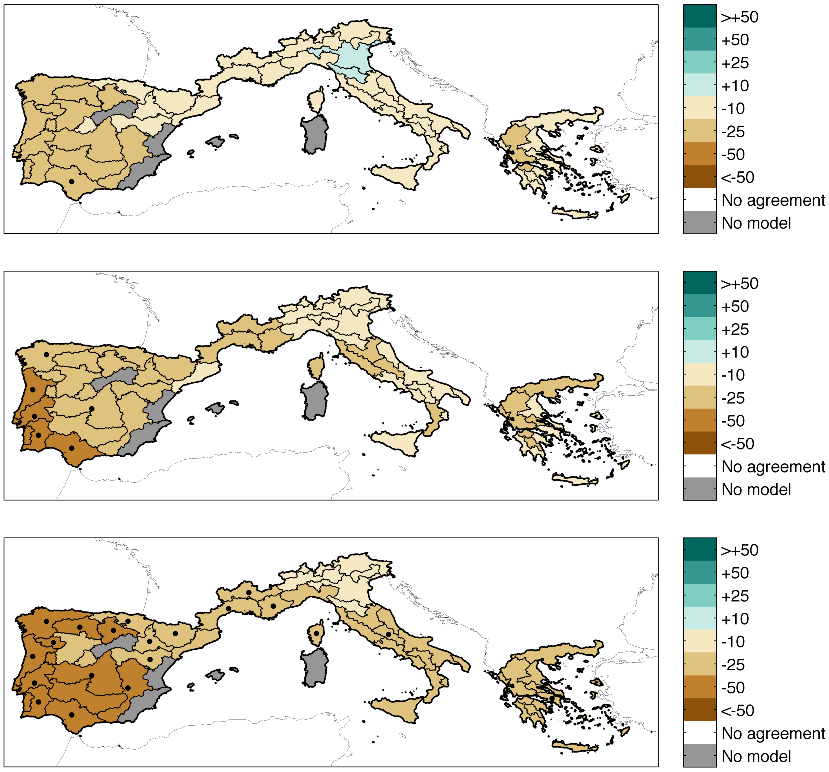

Supplementary Figure 7: Ensemble mean PRE changes (in percentage), with respect to 1971-2000, for the +1.5°C case (first line), the +2°C case (second line) and the +3°C case (third line). Dots indicate areas where at least 50% of the models show a statistically significant change and more than 66% agree on the direction of the change; coloured area (without dots) indicate that changes are small compared to natural variations; white regions (if any) indicate that no agreement between the RCMs is found (similarly to[4]).

Region	Model	RhoIN	RhoOUT
ES01	Y=9.55-0.85 $\cdot SPEI_3(0,8)$	0.52	0.40
ES02	Y=5.21-1.14 $\cdot SPEI_6(0,8)$	0.70	0.66
ES03	Y=9.69-0.72 $\cdot SPEI_3(0,8)$-0.09 $\cdot T$	0.80	0.73
ES04	Y=7.51-1.08 $\cdot SPEI_3(0,8)$-0.11 $\cdot T$	0.85	0.80
ES05	Y=9.83-0.69 $\cdot SPEI_3(0,8)$-0.09 $\cdot T$	0.78	0.65
ES07	Y=7.42-1.26 $\cdot SPEI_3(0,7)$	0.74	0.68
ES08	Y=8.84-0.59 $\cdot SPEI_3(0,7)$	0.50	0.37
ES09	Y=9.30-0.51 $\cdot SPEI_3(0,9)$-0.08 $\cdot T$	0.59	0.47
ES10	Y=9.74-0.48 $\cdot SPEI_6(0,9)$-0.10 $\cdot T$	0.75	0.69
ES11	Y=7.02-0.85 $\cdot SPEI_6(0,9)$	0.50	0.41
ES14	Y=7.04-1.18 $\cdot SPEI_3(0,7)$	0.63	0.51
ES15	Y=6.11-0.88 $\cdot SPEI_3(0,7)$	0.49	0.37
ES16	Y=6.63-0.75 $\cdot SPEI_6(0,9)$-0.10 $\cdot T$	0.62	0.51
ES17	Y=5.77-0.94 $\cdot SPEI_3(0,8)$	0.56	0.47
FR01	Y=7.50-0.79 $\cdot SPEI_3(0,8)$	0.64	0.53
FR02	Y=5.17-1.20 $\cdot SPEI_3(0,8)$	0.71	0.67
FR03	Y=6.49-1.08 $\cdot SPEI_3(0,8)$	0.57	0.46
FR04	Y=9.13-1.06 $\cdot SPEI_3(0,7)$-0.10 $\cdot T$	0.76	0.69
GR01	Y=8.87-0.89 $\cdot SPEI_3(0,9)$-0.06 $\cdot T$	0.71	0.63
GR02	Y=8.66-0.80 $\cdot SPEI_3(0,9)$-0.07 $\cdot T$	0.69	0.64
GR03	Y=9.75-0.87 $\cdot SPEI_3(0,8)$-0.10 $\cdot T$	0.67	0.53
IT02	Y=9.23-0.60 $\cdot SPEI_3(0,8)$	0.67	0.59
IT03	Y=5.77-0.37 $\cdot SPEI_3(0,8)$	0.55	0.46
IT04	Y=9.01-0.58 $\cdot SPEI_3(0,8)$	0.64	0.57
IT05	Y=7.48-0.80 $\cdot SPEI_3(0,8)$	0.72	0.66
IT06	Y=8.56-0.66 $\cdot SPEI_3(0,9)$	0.77	0.72
IT07	Y=8.66-0.75 $\cdot SPEI_3(0,8)$	0.77	0.71
IT08	Y=6.07-1.16 $\cdot SPEI_3(0,8)$	0.74	0.68
IT09	Y=5.44-1.17 $\cdot SPEI_3(0,8)$	0.66	0.59
IT10	Y=7.14-0.90 $\cdot SPEI_3(0,8)$-0.08 $\cdot T$	0.77	0.70
IT11	Y=4.68-0.83 $\cdot SPEI_3(0,8)$	0.57	0.50
IT12	Y=7.56-1.24 $\cdot SPEI_6(0,8)$-0.13 $\cdot T$	0.82	0.76
IT13	Y=8.96-0.62 $\cdot SPEI_3(0,8)$-0.12 $\cdot T$	0.91	0.89
IT14	Y=8.12-0.64 $\cdot SPEI_3(0,8)$-0.09 $\cdot T$	0.66	0.61
IT15	Y=5.41-0.97 $\cdot SPEI_3(0,8)$-0.08 $\cdot T$	0.77	0.71
IT16	Y=5.58-1.22 $\cdot SPEI_3(0,8)$-0.15 $\cdot T$	0.80	0.73
PT01	Y=7.92-0.73 $\cdot SPEI_6(0,9)$	0.54	0.42
PT02	Y=9.38-0.87 $\cdot SPEI_3(0,7)$	0.69	0.63
PT03	Y=10.62-0.74 $\cdot SPEI_3(0,8)$	0.63	0.59
PT04	Y=9.71-0.60 $\cdot SPEI_3(0,8)$	0.59	0.53

Supplementary Table 1: Empirical SPEI-fire models (Eq. 1) for each ecoregion (labelled according to Fig. S4) and the correlation for the reconstruction model (RhoIN: in-sample) and for the leave-one-out cross validation model (RhoOUT: out-of-sample).

Institution	ESM	run	RCM
CLMcom	MOHC-HadGEM2-ES	r1i1p1	CCLM4-8-17
DMI	ICHEC-EC-EARTH	r3i1p1	HIRHAM5
KNMI	ICHEC-EC-EARTH	r1i1p1	RACMO22E
KNMI	MOHC-HadGEM2-ES	r1i1p1	RACMO22E
SMHI	CERFACS-CNRM-CM5	r1i1p1	RCA4
SMHI	ICHEC-EC-EARTH	r12i1p1	RCA4
SMHI	IPSL-CM5A-MR	r1i1p1	RCA4
SMHI	MOHC-HadGEM2-ES	r1i1p1	RCA4
SMHI	MPI-ESM-LR	r1i1p1	RCA4

Supplementary Table 2: EURO-CORDEX RCM models used in this study.

	+1.5°-rcp45	+2°-rcp45	+3°-rcp45	+1.5°-rcp85	+2°-rcp85	+3°-rcp85
CNRM-CM5	2035-2064	2057-2086	–	2029-2058	2043-2072	2066-2095
EC-EARTH	2021-2050	2043-2072	–	2018-2047	2035-2064	2059-2088
HadGEM2-ES	2023-2052	2039-2068	–	2019-2048	2031-2060	2052-2081
MPI-ESM-LR	2019-2048	2042-2071	–	2016-2045	2034-2063	2060-2089
IPSL-CM5A-MR	2010-2039	2026-2055	2067-2096	2009-2038	2025-2054	2046-2075

Supplementary Table 3: Periods of each GCM for the three warming levels considered in this study.

References

1. Ventura, V., Paciorek, C. J. & Risbey, J. S. Controlling the proportion of falsely rejected hypotheses when conducting multiple tests with climatological data. *Journal of Climate* **17**, 4343–4356 (2004).

2. Turco, M. *et al.* On the key role of droughts in the dynamics of summer fires in mediterranean europe. *Scientific reports* **7** (2017).

3. Metzger, M., Bunce, R., Jongman, R., Mücher, C. & Watkins, J. A climatic stratification of the environment of europe. *Global ecology and biogeography* **14**, 549–563 (2005).

4. Tebaldi, C., Arblaster, J. M. & Knutti, R. Mapping model agreement on future climate projections. *Geophysical Research Letters* **38** (2011).
